# Associations between parenting strategies and BMI percentile among Latino children and youth with intellectual and developmental disabilities

**DOI:** 10.3389/fped.2023.1189686

**Published:** 2023-07-27

**Authors:** Sandy Magaña, Vanessa L. Errisuriz, Amy Pei-Lung Yu, Nazanin Heydaria, Weiwen Zeng, Mansha Mirza, Sandra Vanegas, Stephany Brown, Deborah Parra-Medina, Yolanda Suarez-Balcazar

**Affiliations:** ^1^ Texas Center for Disability Studies, Steve Hicks School of Social Work, University of Texas at Austin, Austin, TX, United States; ^2^ Latino Research Institute, University of Texas at Austin, Austin, TX, United States; ^3^ School of Social Work, University of Texas Rio Grande Valley, Edinburg, TX, United States; ^4^ Department of Occupational Therapy, Department of Disability and Human Development, University of Illinois at Chicago, Chicago, IL, United States

**Keywords:** healthy weight, intellectual and developmental disabilities, Latino, obesity, parenting strategies

## Abstract

**Introduction:**

Maintaining healthy weight is a challenge for all children, and particularly for children with IDD compared to nondisabled children and for Latino children compared to non-Latino White children. Parenting practices related to food intake and physical activity have been found to be important in maintaining children's weight. In this study, we describe the prevalence of overweight and obesity status among Latino children with IDD and their maternal caregivers and determine the relationship between food and physical activity parenting practices and childhood obesity among Latino children with IDD.

**Methods:**

We interviewed 94 Latino parent/child dyads and collected information about parenting practices, home environment, and parent and child height and weight using standardized measures. Parent body mass index (BMI) and child BMI percentile were calculated from height and weight.

**Results:**

The combined overweight/obesity status for children in our sample was high (60.3%) compared to national rates among nondisabled Latino children (56%) and non-Latino White children with autism (37%). Contrary to research on nondisabled children, we found that greater parental use of controlling dietary strategies was associated with lower BMI percentile in Latino children with IDD. These findings may be indicative of the fact that children with IDD tend to have unique dietary behaviors that warrant more disability and culturally sensitive strategies.

**Discussion:**

Our findings suggest that overweight and obesity is especially prevalent for Latino children with IDD and that more research is needed on family factors that promote health in Latino families of children with IDD.

## Introduction

One of the most common non-communicable health problems is overweight and obesity. The World Health Organization (WHO) defines overweight and obesity as conditions with excessive accumulation of body fat that presents a risk to health (1). Recent projections from the World Obesity Federation suggest that by 2060, approximately 91% of children and adults in the United States will be overweight or obese (2). The economic costs of overweight and obesity in the United States are projected to increase to $2.622 trillion by 2060 (2). Systematic reviews find that children and youth who are overweight or obese are more likely to develop and experience cardiovascular illness and diabetes, asthma, musculoskeletal discomforts, psychological distress, anxiety, and depressive symptoms (3–5), and that these effects can be long-lasting and persist into adulthood (6, 7). Furthermore, longitudinal studies find that children and youth who are overweight or obese face significant levels of stigma, achieve lower levels of education, and report lower quality of life than children and youth who are not overweight or obese (8).

Within the United States, Latino^1^ children and youth (aged 2–19 years) are more likely to be overweight or obese than White children, with obesity rates reported as high as 26.2% among Latino children compared to 16.6% among White children (9–12). Moreover, children and youth with intellectual and developmental disabilities (IDD) have also been identified as more likely to be overweight or obese than children without IDD, ranging from 33.5% to 47.0% of children with IDD (13). These data are striking as Latino children and children with IDD experience significant obstacles in accessing a primary care provider, specialty health care services, and family-centered care (14, 15). Further disparities arise when we consider the intersectional identities of Latino children with IDD, as they may face substantial barriers to accessing health and education services and supports (14, 16).

Body fat composition is influenced by environmental factors affecting caloric surplus and deficit that result from food intake and caloric expenditure through physical activity (17). Family environment, particularly parenting practices related to food and physical activity, plays an important role in children's dietary and physical activity behaviors (18). Food parenting practices (FPP) are intentional or unintentional, food-specific actions or behaviors used by parents for child-rearing purposes that affect their child's eating-related attitudes, beliefs, or behaviors (19). Among non-disabled children, FPP are strongly associated with diet and weight. Parents who utilize factors that emphasize structure, warmth, and responsiveness (e.g., parent encouragement and modeling healthy behavior) and use family rules, limit-setting, and monitoring are more likely to have children who eat healthier foods (19–23). Conversely, parents who use more controlling FPP, such as feeding restrictions, overfeeding, using foods as rewards, and pressure to eat, have been linked to poorer diet and obesity development (24–27). Samples used in these studies were of predominantly White families and children. A study on food intake, FPP, and acculturation among Latino children and families found that immigrant mothers used more controlling and less indulgent feeding practices compared to Latina mothers born in the United States (28) and greater maternal acculturation to the United States was associated with higher unhealthy food consumption and higher rates of obesity among Latino children in a different study (29).

Parents may be particularly underprepared in FPP with their children and youth with IDD. Parents of children with IDD often face unexpected feeding issues as opposed to parents of nondisabled children (30, 31). Feeding issues are especially marked for children with autism spectrum disorder (ASD), who tend to have more restricted diets due to food selectivity, texture sensitivities, and specific mealtime expectations (32, 33). In addition, children with ASD may also be affected by medication side effects, sensory issues, specialized or focused behaviors, and gastrointestinal symptoms (34, 35). Therefore, parents of children with ASD are likely to be more involved in monitoring their child's diet and are more vigilant about their child's dietary behaviors to ensure that their child is getting sufficient nutrients (36, 37). Children with other types of IDD, such as intellectual disability or Down syndrome (DS), have different challenges with respect to feeding. For some children with DS, eating can be a challenge due to chewing and swallowing difficulties (38). One study found a notable tendency to overfill mouths and cheeks with food without swallowing in a sample children with DS (39). While children and youth with DS have a greater tolerance for various food textures than children with ASD, this same study found a preference for crispy and oily foods and a dislike for brittle and gooey foods leading to greater consumption of salty and cheesy products (39). Children with DS also demonstrate similar patterns of dietary behaviors to the general pediatric population with excessive consumption of protein and saturated fat, and preferred bread, pasta, fruit juices, meat, and cold cuts (38). In combination, these factors can lead to a less nutritious diet in this group. The challenges children with DS and IDD experience with eating may influence the types of strategies or practices parents use to impact their child's eating behaviors.

Research has demonstrated racial/ethnic differences in the influence of FPP on children's eating and weight. For instance, pressure to eat and restrictive feeding practices are more commonly observed among Latino, non-Latino Black, and Asian parents compared to non-Latino White parents (40–43). However, the results of studies conducted among Latino children have been mixed. Hughes et al. (44) found that Latino parents tend to be more indulgent of their children's diet (i.e., characterized by warmth and acceptance in combination with a lack of monitoring of the child's behavior) and that such behavior was associated with higher child BMI. In contrast, a recent study using data from the Hispanic Community Children's Health Study/Study of Latino Youth (SOL Youth) found that children (ages 8–16 years) of parents who used controlling FPPs vs. indulgent FPPs were 1.75 times more likely to consume a diet that is high in calories (45). Although FPP are widely examined among typically developing children and youth, research on children and youth with IDD has been scant. There is even less research that has examined FPP and the intersection of race and disability.

Parenting practices can also influence children's engagement in physical activity. Research shows that when mothers exercise certain levels of control and enforce behavioral expectations, children are likely to spend more time engaging in physical activity and spend less time being sedentary (46, 47). For Latino children, it appears that parent involvement has a positive effect on the children's physical activity. One integrative review found that Latino children's engagement in physical activity was facilitated when parents monitored their child's behaviors, encouraged, or prompted their child to be more physically active, modeled active behaviors, offered logistic support for physical activity participation, and presented rewards and reinforcement for being physically active (48). However, Latino families of children with disabilities often mention barriers to engaging in physical activity (49, 50). These barriers include inclement weather, high cost, lack of time, neighborhood safety, and lack of space to exercise at home (50). Moreover, Latino families may be more likely to live in neighborhoods with limited access to affordable recreational facilities and healthy food options, which makes it difficult to maintain physical activity and a healthy diet routine (51–53).

Research on physical activity and parenting practices is very limited for children and youth with IDD. A systematic review of 30 studies demonstrated that children with IDD are less likely to engage in physical activity than children without disabilities (54). In an effort to promote physical activity, parents of children and youth with IDD may utilize a variety of parenting practices. A systematic review on children with disabilities, broadly speaking, identified three dimensions of parenting that were significantly and positively associated with physical activity behaviors in children with disabilities: participating in physical activity interventions, parental support (e.g., providing transportation, offering encouragement) and parents' perceived importance of their child engaging in physical activity (55). In addition to parent support, parents' perceived competence of their child's physical ability has also emerged as a key factor in promoting physical activity behaviors of children with disabilities (56). Some studies have identified barriers to engagement in organized physical activity for children and youth with disabilities. According to one study, these barriers include low socio-economic status, parents' and siblings' non-participation in physical activity, and being enrolled in special education vs. a mainstream school (57). Thus, families and children at the intersection of race and disability might face compounded barriers to physical activity engagement.

The physical home environment is an important determinant of child diet, physical activity, and obesity. Previous research indicates that children who live in households characterized by greater availability of unhealthy foods, fewer fruits, and vegetables, fewer sports/recreational equipment items, and more media equipment are at high risk for obesity. While high rates of childhood overweight/obesity in the Latino community are often attributed to cultural influences, attitudes, and beliefs (58), most Latino parents do recognize the importance of children eating healthy foods, fewer sweets, and being physically active (48). Yet, due to structural and economic barriers, they may struggle to provide healthy foods and find time and space to engage in physical activity with their children.

To date, there is limited research on Latino children with IDD related to weight and its associated factors. The influence of family context is understudied but is substantially important given this population's higher dependence on parents, siblings, and caregivers to accommodate their food intake and physical activity (59, 60). As the prevalence of obesity among Latino children with IDD continues to rise (61), examining the associations between food and physical activity parenting practices, the home food and physical activity environment, and obesity are warranted.

The aims of the current study are to (1) describe the prevalence of overweight and obesity status among Latino children with IDD and their maternal caregivers; and (2) determine the relationship between food and physical activity parenting practices, home food and physical activity environment, and childhood obesity among Latino children with IDD.

## Methods

### Study design and recruitment

The present study is based on data collected for a larger, cross-sectional study that investigated the health behaviors (e.g., diet, physical activity) and lifestyle factors (e.g., socioeconomic status, home environment) contributing to overweight and obesity among Latino children with intellectual and developmental disabilities (IDD) and their maternal caregivers. Mothers were selected for the study as they are typically the primary caregiver of children with IDD in Latino families (62). Research staff recruited caregiver-child dyads from two urban cities in the United States, both of which were characterized by large Latino populations (63). Institutional review boards of universities at both study sites approved the study. We formed a Community Advisory Board (CAB) at each site, consisting of researchers, representatives of local Latino-serving non-profit organizations, parents of a child with IDD, and disability self-advocates. The CABs provided ongoing input to the research team related to study design, recruitment, evaluation, and dissemination. Community agencies that serve the target population assisted with recruitment by sharing recruitment materials (i.e., a Spanish and English bilingual informational flyer) with Latino families through their websites, newsletters, email listservs, and social media (e.g., Facebook). To increase recruitment, research staff also delivered community outreach presentations at events such as local community fairs and parent support groups.

### Participants

Families expressed interest in the study by filling out an online study interest form. After receiving the interest forms, research staff conducted eligibility screenings with families by phone. Eligibility criteria included: (1) the caregiver identified as a Latina mother (or other Latina female primary caregiver with custody of the child); (2) the caregiver had a child between 6 and 17 years of age; (3) the focal child had a diagnosis of autism spectrum disorder (ASD), Down syndrome (DS), and/or intellectual disability (ID); and (4) the focal child was able to walk since we also collected accelerometer data (accelerometers have not been well tested with children who are unable to walk without a device). Families with more than one child with IDD were encouraged to include all eligible children in the study.

For eligible families, trained bilingual research staff obtained verbal consent from the caregiver, parental consent for child participation, and child assent from each child. Between July 2020 and December 2022, we completed interviews with 94 Latino caregiver-child dyads via three, 60- to 75-minute phone or video calls over the course of 4–6 weeks. Seven families had more than one eligible child bringing the total number of enrolled children with IDD to 101. During the interviews, research staff administered survey questions in English or Spanish depending on preference, including demographics and caregiver-child health, health behaviors, quality of life, socioeconomic conditions, home environment, as well as height and weight data to calculate body mass index (BMI). Families could choose to complete assessments either in Spanish or English. Each family received a $25 gift card voucher as compensation for each interview completed, earning up to $75 for all three interviews.

Table 1 presents caregiver and child demographics of our study sample. Sixty-four families chose to complete the interviews in Spanish and the remaining 30 in English. On average, Latina caregivers were 42.9 (SD = 6.2) years old with a range from 30 to 58. Most of them were from lower socioeconomic backgrounds—about 60% had an annual household income of $35,000 or below; about half said they owned a house/apartment (45%), were uninsured (43%), and had high school or less education (48%); only 27% said they were employed/self-employed while others identified either as full-time caregivers (36%) or were unemployed/unable to work (37%). Most caregivers were married/partnered (82%) and born outside of the US (81%). For children with IDD, they were on average 10.8 (SD = 3.3) years old. Most of them were boys (71%), born in the US (95%), had insurance of any kind (96%), and had a primary diagnosis of autism (78%).

**Table 1:** Baseline demographic characteristics.

Characteristics	Mother (*N* = 94)	Characteristics	Child with IDD (*N* = 101)
Mean age (SD)^a^	42.9 (6.2)	Mean age (SD)	10.8 (3.3)
	*n* (%)		*n* (%)
Annual household income^a^		Male	72 (71.3)
≤$35,000 USD	52 (57.8)	Place of birth	
>$35,001 USD	38 (42.2)	US-born	96 (95.0)
Maternal education^a^		Foreign-born	5 (5.0)
High school or less	40 (47.6)	Insurance status	
Some college or more	44 (52.4)	Uninsured	4 (4.0)
Employment status^a^		Insured, any kind	97 (96.0)
Employed/self-employed	23 (27.4)	Primary diagnosis^a^	
Full-time caregiver	30 (35.7)	ASD	79 (78.2)
Unemployed/unable to work	31 (36.9)	ID/DS	22 (21.8)
Married/partnered^a^	68 (81.9)		
Foreign-born^a^	68 (81.0)		
Insurance status^a^			
Uninsured	36 (43.4)		
Insured, any kind	47 (56.6)		
Home ownership^a^			
Own a house/apt	37 (44.6)		
Rent/other arrangement	46 (55.4)		
Language preference			
Spanish	64 (68.1)		
English	30 (31.9)		
BMI and weight status^b^	Mother (*N* = 94)	BMI and weight status^b^	Child with IDD (*N* = 93)
Mean BMI (SD)	30.2 (6.7)	Mean BMI percentile (SD)^a^	75.2 (31.7)
Weight status	*n* (%)	Weight status^a^	*n* (%)
Under weight	4 (4.3)	Underweight	9 (9.7)
Normal weight	16 (17.0)	Normal weight	28 (30.1)
Overweight	24 (25.5)	Overweight	22 (23.7)
Obese	50 (53.2)	Obese	34 (36.6)

SD, standard deviation; IDD, intellectual and developmental disabilities; ASD, autism spectrum disorder; ID, intellectual disability; DS, Down syndrome; BMI, body mass index.

^a^ Sample sizes varied due to missing data. Data reported are for valid Ns and percentages.

^b^ Adult weight status was categorized using raw caregiver BMI data: underweight (<18.5), normal weight (18.5–24.9), overweight (25.0–29.9), and obese (≥30.0). Child weight status was categorized using child BMI percentile data: underweight (<5.0), normal weight (5.0–84.9), overweight (85.0–94.9), and obese (≥95.0).

Dichotomous variables: caregiver—annual household income (0 ≤ $35,000, 1 > $35,000), education (0 = high school or less, 1 = some college or more), employment (0 = not employed/full-time caregiver, 1 = employed/self-employed), marital status (0 = not married, 1 = married/partnered), country of origin (0 = foreign-born, 1 = US-born), insurance status (0 = uninsured, 1 = insured, any kind), and home ownership (0 = rent/other arrangement, 1 = own a house/apartment). Child—gender (0 = female, 1 = male), place of birth (0 = foreign-born, 1 = US-born), insurance status (0 = uninsured, 1 = insured, any kind), and primary diagnosis (0 = ID/DS, 1 = ASD).

### Measures

#### Demographics

Questions for caregivers included age, country of origin, education, employment, marital status, annual household income, insurance status, and home ownership. We also collected child demographics based on caregiver-reported data, including child age, gender, place of birth, insurance status, and IDD primary diagnosis.

#### Brief autism mealtime behaviors inventory (BAMBI)

To capture mealtime behaviors specific to children with IDD, we used the BAMBI, a 14-item questionnaire that asks parents to indicate how often their child exhibited negative (e.g., crying, screaming, and being disruptive during mealtimes) and positive (e.g., open to trying new foods and remained seated at the table while eating) behaviors (64). Responses are scored on a 5-point Likert-type scale ranging from 1 “never occurs” to 5 “always occurs”. Positive behavior items are reverse-coded. A total frequency score was calculated by summing all 15 items, with higher scores reflecting more mealtime behavioral problems. As discussed earlier, children with ASD may have different mealtime behaviors when compared to those with DS or ID. We thus used the BAMBI scores as a covariate which we will discuss further in the analysis. The Cronbach's alpha is 0.79 for BAMBI for the current sample.

#### Home environment

We examined home food (HFE) and physical activity (HPAE) environment using items adapted from the Home Health Environment survey developed by Boles et al. (65). For home food environment, caregivers reported how often healthy (e.g., raw fruits, vegetables, and low-fat crackers) and unhealthy foods (e.g., chocolate candy, cookies, and regular sodas) were available to their children at home on a 5-point Likert-type scale from 1 “never” to 5 “always”. For home physical activity environment, caregivers reported how often their children used certain physical activity equipment (e.g., bikes, jump rope, and active video games) available to them at home on a 5-point Likert-type scale from 1 “not available/don’t use at all” to 5 “once a week or more”. Items for healthy foods, unhealthy foods, and physical activity equipment were summed to calculate index scores. The Cronbach's alphas are 0.32 for the healthy food index, 0.75 for the unhealthy food index, and 0.65 for the physical activity equipment index for the current sample. Due to its low internal consistency, the healthy food index was excluded from the analysis.

#### Parenting strategies for eating and activity scale (PEAS)

To assess strategies that caregivers use to impact child diet and physical activity at home, we used the 26-item PEAS, developed to be culturally appropriate for Latino families (66). Based on 5-point Likert-type responses ranging from 0 “never” to 4 “very often”, the PEAS measures five parenting strategies including three for diet (limit setting, monitoring, and control) and two for physical activity (monitoring and reinforcement). Items for each parenting strategy for diet (PEAS-diet) and physical activity (PEAS-PA) were summed to calculate index scores. The Cronbach's alphas are 0.81 for the PEAS-diet and 0.80 for the PEAS-PA for the current sample.

#### Body mass index (BMI)

Due to the COVID-19 restrictions, we relied on caregiver-report data. Caregiver and child heights (in inches) and weights (in pounds) were obtained. Specifically, mothers were first asked to report their own weight and height. They were then asked if they had a scale and tape measure available at home. If the response was “yes,” they were asked to conduct the child measurements in real-time. Otherwise, mothers were asked to recall their child's weight and height data from their last pediatric visit. Caregiver BMI was calculated by dividing weight in pounds by height in inches squared and multiplying by a conversion factor of 703. A BMI of 25.0–29.9 in adults is considered overweight, and a BMI of 30.0 or higher is considered obese (67). For children, we used BMI age- and sex-adjusted growth charts, which are known to provide a reliable indicator of excess adiposity in children (68). Child BMI at the 85th percentile is considered overweight and 95th percentile is considered obese.

### Data analysis

Data analyses were conducted using the Statistical Package for the Social Sciences (SPSS), version 27 (IBM Corp, Armonk, NY). Means, standard deviations, and frequencies for demographic characteristics of the caregiver-child dyads were calculated (see Table 1). Standardized measures were computed based on their respective scoring schemes. Distributional analysis was done to ensure that the data were normally distributed and free from errors. There were missing data throughout the raw dataset because of participant non-response (e.g., caregivers were concerned about providing certain personal information). Missing data ranged from 0 (child age) to 17% (marital status). Overall, missing data accounted for 7% of the overall dataset and diagnostics showed no patterns for the missing data. To address the missingness so that the full sample could be included in analyses, multiple imputation was conducted with the MI command in SPSS. Specifically, we used the default setting which ran five iterations using the automatic method assuming missing-at-random (MAR). Model constraints were set based on original data (e.g., child BMI percentile was limited to 0–100). SPSS generated pooled results through the iterative simulations in which missing values were replaced by plausible estimates (69). The pooled results thus represent the least-biased estimates for the study parameters and were used in the analysis.

Using caregiver BMI and child BMI percentile derived from caregiver-report weight and height data, we calculated the numbers and percentages of caregivers and children in our sample that were overweight or obese to compare with national prevalence rates of obesity among Latino children and adult women (Aim 1). To examine the relationships between home food and physical activity environment, parenting practices, and child BMI percentile (Aim 2), we conducted a series of hierarchical linear regression models with child mealtime behaviors (BAMBI) as a covariate throughout. Home environment (i.e., unhealthy food and physical activity equipment indices) and parenting strategies were entered as the first block in the hierarchical regression model to examine the crude relationships between these variables and child BMI percentile while accounting for child mealtime behaviors. Caregiver-child factors were entered as the second block to examine the effects of home environment and parenting strategies net of socioeconomic and demographic variables. Four child variables were excluded in the second block—child age and gender were excluded because they were already adjusted when calculating child BMI percentile (see CDC, https://www.cdc.gov/healthyweight/bmi/calculator.html); child place of birth and insurance status were excluded due to a lack of variability. Subsequently, caregiver-child factors entered in the second block included caregiver age, BMI, country of origin, education, employment, marital status, annual household income, insurance status, home ownership, and child primary diagnosis. The model fit was examined using the proportions of the variance explained in the dependent variable (i.e., *R*^2^ and *R*^2^ change).

## Results

### Caregiver-report BMI, child BMI percentile, and weight statuses

On average, caregiver self-report BMI was 30.2 (SD = 6.7) with a range from 12.4 (severely underweight) to 52.9 (severely obese) in our study sample. More than half (53%) of the caregivers met criteria for adult obesity (BMI ≥30.0) and 26% were overweight but not obese for a combined 79% with overweight/obesity status. Caregiver-report child BMI percentile was on average 75.2 (SD = 31.7) and ranged from 0.1 (severely underweight) to 99.8 (severely obese). About 37% of the children with IDD met criteria for obesity (BMI percentile ≥95.0), and 24% were assessed to be overweight but not obese. Combined overweight and obesity status among the children was 60.3%. Caregiver-report BMI and child BMI percentile were significantly correlated (Pearson's *r* = 0.22, *p* = 0.04). Among the 50 caregivers who were reported to be obese, 21 reported their children were also obese.

### Home environment and parenting strategies for eating and physical activity

Table 2 shows the characteristics of, and correlations between the home environment and parenting strategies subscales. On average, limit setting for diet (*M* = 13.4, SD = 5.6) and reinforcement for physical activity (*M* = 12.0, SD = 4.5) had higher scores indicating greater use by Latina caregivers. The correlation matrices show that physical activity monitoring was strongly correlated with all other parenting strategies. Additionally, limit setting and monitoring for diet were strongly correlated with each other. For home environment subscales, unhealthy food index was significantly correlated with physical activity monitoring, while physical activity equipment index was significantly correlated with physical activity reinforcement and unhealthy food index. No significant correlations were found among other parenting strategies subscales.

**Table 2:** Characteristics of and correlations between home environment and parenting strategies subscales.

Variables	*N*	Mean	SD	1	2	3	4	5	6	7
1. Diet—Limit setting	101	13.4	5.6	–						
2. Diet—Monitoring	101	10.7	6.1	0.54***	–					
3. Diet—Control	101	7.8	4.9	0.09	0.18	–				
4. PA—Monitoring	101	8.7	5.7	0.40***	0.57***	0.39***	–			
5. PA—Reinforcement	101	12.0	4.5	0.14	0.05	0.05	0.33**	–		
6. Unhealthy food index	94	19.4	5.3	−0.19	−0.16	−0.04	−0.22*	−0.17	–	
7. PA equipment index	94	19.6	6.9	0.03	0.07	0.05	0.11	0.22*	0.27**	–

SD, standard deviation; PA, physical activity.

* *p *< 0.05, **p < 0.01, ***p < 0.001.

### Hierarchical regression models

Table 3 shows the statistics of the two hierarchical regression models. In model 1, home environment and parenting strategies variables were entered as the first block while only child mealtime behaviors were accounted for. Results show that (1) controlling in parenting strategies for diet significantly predicted lower child BMI percentile (*t *= −2.49, *p *= 0.01), (2) monitoring in parenting strategies for physical activity significantly predicted higher child BMI percentile (*t *= 2.31, *p *= 0.02). The model fit statistics show that home environment and parenting strategies explained 15% of the variance (*R*^2^ = 0.15 *p *= 0.04) in child BMI percentile.

**Table 3:** Results of hierarchical regression models for child BMI percentile.

Independent Variables	Model 1		Model 2	
	*t*	*p-*values	*t*	*p-*values
Home environment				
Unhealthy food index	−0.14	0.89	−0.37	0.71
PA equipment index	−0.73	0.47	−0.60	0.55
Parenting strategies				
Diet—Limit setting	1.61	0.11	1.58	0.11
Diet—Monitoring	−1.30	0.19	−1.32	0.19
Diet—Control	−2.49	0.01*	−2.47	0.02*
PA—Monitoring	2.31	0.02*	2.34	0.02*
PA—Reinforcement	−1.29	0.20	−0.59	0.56
Caregiver-child factors				
Caregiver age			−0.27	0.79
Caregiver BMI			1.17	0.25
Caregiver country of origin			−0.51	0.61
Caregiver education			−1.14	0.26
Caregiver employment			1.44	0.15
Caregiver marital status			1.50	0.14
Caregiver insurance status			0.70	0.49
Annual household income			−0.69	0.50
Home ownership			−0.23	0.82
Child IDD primary diagnosis			−2.15	0.04*
	*R* ^2^	*p-*value	*R* ^2^	*p-*value
* *	0.15	0.04*	0.28	0.02*

PA, physical activity; BMI, body mass index; IDD, intellectual and developmental disabilities.

Dichotomous variables: caregiver—country of origin (0 = foreign-born, 1 = US-born), education (0 = high school or less, 1 = some college or more), employment (0 = not employed/full-time caregiver, 1 = employed/self-employed), marital status (0 = not married, 1 = married/partnered), insurance status (0 = uninsured, 1 = insured, any kind), annual household income (0 ≤ $35,000, 1 > $35,000), and home ownership (0 = rent/other arrangement, 1 = own a house/apartment). Child—IDD primary diagnosis (0 = ID/DS, 1 = ASD).

* *p *< 0.05, **p < 0.01, ***p < 0.001.

In model 2, caregiver-child factors were further entered to examine the net effects of home environment and parenting strategies. Consistent with model 1, both controlling for diet (*t *= −2.47, *p *= 0.02) and monitoring for physical activity (*t *= 2.34, *p *= 0.02) remained significant predictors of child BMI percentile. Among all caregiver-child factors entered, only the child's primary diagnosis of ASD significantly predicted lower child BMI percentile (*t *= −2.15, *p *= 0.04). In model 2, 28% of the variance (*R*^2^ = 0.28, *p *= 0.02) in child BMI percentile were explained which represents a 13% (*R*^2^ change = 0.13) increase from model 1.

## Discussion

In the present study, we examined prevalence of overweight and obesity status in a multisite sample of Latino children with IDD and correlates of the children's Body Mass Index (BMI) percentile with a focus on parenting strategies. Thirty-seven percent of the children in our study met criteria for obesity status, and 24% met criteria for overweight status (not obese). The combined overweight/obesity status for children in our sample was 60.3%. This is comparatively higher than overweight/obesity status among a representative sample of nondisabled Mexican American children who were reported to be 55.5% overweight/obese (70); and compared to a meta-analysis estimate of children with autism, which was reported to be 37% overweight/obese (71). Caregivers in our sample also had similarly high rates of overweight/obesity status (79%) compared to a representative sample of women assessed in 2016 at 68% (72). These findings are a call to action for more research into the determinants and consequences of obesity and overweight status in racial/ethnic minoritized children with IDD and their family caregivers.

To investigate further, we examined correlates and predictors of child BMI percentile with a focus on home food and physical activity environment and parenting strategies for eating and physical activity. Contrary to prior findings, there were no effects of the presence or absence of healthy foods (73, 74) or the presence of sports equipment (75) on child BMI percentile. This discrepant finding could be related to limitations with activities of daily living (ADL) independence among children with IDD. Studies show that children and youth with IDD experience significant difficulties with performing ADL, thereby restrict their participation in everyday activities that their age peers can perform independently (76, 77). Such activities might include serving themselves snacks available at home or initiating physical activities using available home equipment on their own. Covert changes in the home environment, such as purchasing healthy foods and making exercise equipment readily available, are contingent on the child's ability to notice these changes and initiate dietary and physical activity behaviors (78). For children with IDD, this type of covert control might be insufficient in isolation without active guidance and direction from caregivers.

With respect to parenting practices, we found that greater parent use of controlling strategies for child diet was associated with lower child BMI percentile. This finding is in contrast to what has been reported in previous research of non-disabled children, which finds that greater use of controlling behaviors is associated with higher BMI percentile (24–27). Research in the Latino community has similarly found an association between greater controlling strategies by the parents and higher caloric intake of the children (45). However, a study on immigrant parents finds that they use more controlling strategies with their children, which are not always associated with negative outcomes (28). Mothers in our study were primarily immigrant parents which may partially explain our findings with respect to use of controlling strategies (28, 29). Additionally, our findings suggest that parents of children with IDD may need to use different strategies than parents of nondisabled children. Children with IDD tend to have unique dietary behaviors related to oral-motor difficulties and food sensitivities and lesser autonomy in self-regulating dietary intake (32, 33). In such situations, parents might feel compelled to exercise greater control and vigilance of their child's dietary intake, which may lead to better outcomes for children with IDD. Consistent with theory, a recent study with 440 participants with IDD found that restricting access to unhealthy foods and sedentary behaviors was associated with children consuming less fried food and less sweetened drinks (79).

Whether parents used monitoring strategies for diet intake was not related to child BMI percentile. However, we found that greater monitoring for physical activity was related to a higher child BMI percentile. This finding contrasts with previous research on children with IDD who were more likely to be physically active when parents monitored and encouraged their child's physical activity engagement (55). Families of children with disabilities, especially from racial/ethnic minority backgrounds, face significant environmental barriers that restrict their ability to find opportunities for their children that are accessible and accommodating of their child's motor and sensory needs (51–53). Thus, environmental barriers might explain why parents' monitoring behaviors might have the opposite effect on their child's BMI in the absence of real and feasible opportunities for physical activity participation. Further research is needed to isolate the individual and combined effects of parenting strategies and neighborhood environments for Latino children with IDD.

We examined the relationships of demographic variables to BMI percentile in our second regression model to determine if they change or enhance the findings from model 1. We found that the findings regarding parenting practices were retained, and the final model was enhanced by identifying an additional relationship. We found that children with IDDs other than autism had higher BMI percentiles. This finding might be related to greater restrictive dietary habits among children with autism (32, 33) while children with IDD may be more prone to dietary excess as suggested by one study (38).

### Limitations and future directions

There are a few important limitations to note. First, it is a cross-sectional study which does not allow us to determine the direction of effects. Future research should consider longitudinal designs. Secondly, this is a convenience sample from Illinois and Texas with predominantly Mexican American Latino populations, which may not represent all Latino families of children with IDD in the US. Another limitation is that this study only included maternal caregivers. Future research may examine the role and influence of fathers, grandparents, and/or siblings in shaping child health behaviors. One study found that Latino fathers are more likely to engage in male and female stereotyped activities (e.g., playing games, cooking, reading) than their White counterparts (80) demonstrating the importance of including fathers in future studies. In the present study, caregiver BMI and child BMI percentile were assessed based on caregiver-report due to the COVID-19 restrictions. Past literature has shown that the use of self-report weight and height data often lead to underestimated BMI (81). We also note that while BMI is widely used in public health, this measure does not capture variation in fat distribution, muscle, and bone density. Furthermore, BMI is not stratified by racial and sex differences in body composition (82). Particularly for children, the BMI does not reflect the onset of puberty, which can be impacted by race, nutrition, genetic and environmental factors (83). Thus, to better assess weight status, future studies should seek to include measures of different body composition metrics such as abdominal adiposity, waist circumference, and percent body fat. Lastly, it is important to acknowledge that all measures used in this study were based on self-report. Subjective measures of health are important in that they convey the experience of wellness among participants. However, future research may seek to corroborate findings by using different subjective measures of health combined with objective data.

Despite the limitations, the present study is significant and innovative in that it considers the family context and gathers information from both caregiver and child. Furthermore, it is among the first to examine the family context in Latino families with children with IDD. These families are particularly subject to cost, safety, and transportation barriers to community resources supporting nutrition and physical activity (51–53). Future research should examine more closely the relationship between parent controlling diet behaviors and healthy weight to determine how this practice should be supported in intervention development for Latino families of children with IDD. Future studies may also examine the mediating role of disability-sensitive attitudes and skills regarding engaging children with disabilities in physical activity on the effects of physical activity (as well as healthy food) environment on child outcomes. This is especially pressing for Latino families because their participation in health promotion programs is often limited by a lack of cultural sensitivity in program recruitment and content (80). Given that prior research has found that children with disabilities are less likely than their non-disabled peers to engage in physical activity (54) and their parents report more difficulties with feeding their children (30, 31), it is likely that this disparity of participation in health promoting programming is particularly pronounced for Latino families of children with disabilities. Our findings may have implications for future culturally tailored interventions for Latino families of children with IDD. There are some interventions that have been culturally tailored for Latino families and children (84, 85). However, future research will need to examine how to incorporate parenting strategies that may be unique to Latino parents of children with IDD into interventions and programs.

### Conclusions

The present study examined the association between parenting strategies with body composition in a population that is often understudied—Latino children with IDD. Our findings establish that Latino children with IDD have worryingly high rates of obesity and add to the understanding of the associations between food and physical activity parenting practices, the home food and physical activity environment, and obesity in the Latino community. The present study is a significant and innovative exploratory work that prompts further examination of family factors that promote health in Latino families with children with intellectual and developmental disabilities.

## Data Availability

The raw data supporting the conclusions of this article will be made available by the authors, upon request.

## Footnotes

^1^We understand that terminology preferences often vary. Some prefer to be identified as Latino, while others prefer Latine or Latinx. Given the lack of consensus, we have chosen to honor the preferences of our research participants who expressed greater comfort and familiarity with being identified as “Latino”. “Latina” will be used when referring to females.

## References

[1] World Health Organization. Obesity. Obesity. Available from: https://www.who.int/health-topics/obesity (Cited 2023 Mar 14).

[2] Okunogbe A Nugent R Spencer G Powis J Ralston J Wilding J. Economic impacts of overweight and obesity: current and future estimates for 161 countries. BMJ Glob Health. (2022) 7(9):e009773. 10.1136/bmjgh-2022-009773 36130777 PMC9494015

[3] Reilly JJ. Descriptive epidemiology and health consequences of childhood obesity. Best Pract Res Clin Endocrinol Metab. (2005) 19(3):327–41. 10.1016/j.beem.2005.04.002 16150378

[4] Russell-Mayhew S McVey G Bardick A Ireland A. Mental health, wellness, and childhood overweight/obesity. J Obes. (2012) 2012:281801. 10.1155/2012/281801 22778915 PMC3388583

[5] Yanovski JA. Pediatric obesity. An introduction. Proc Am Univ Symp Child Obes Cogn. (2015) 93:3–12. 10.1016/j.appet.2015.03.028 PMC4546881 25836737

[6] Kelsey MM Zaepfel A Bjornstad P Nadeau KJ. Age-related consequences of childhood obesity. Gerontology. (2014) 60(3):222–8. 10.1159/000356023 24434909

[7] Singh GK Kogan MD Van Dyck PC Siahpush M. Racial/ethnic, socioeconomic, and behavioral determinants of childhood and adolescent obesity in the United States: analyzing independent and joint associations. Ann Epidemiol. (2008) 18(9):682–95. 10.1016/j.annepidem.2008.05.001 18794009

[8] Sutaria S Devakumar D Yasuda SS Das S Saxena S. Is obesity associated with depression in children? Systematic review and meta-analysis. Arch Dis Child. (2019) 104(1):64–74. 10.1136/archdischild-2017-314608 29959128

[9] Harrington S. Overweight in Latino/Hispanic adolescents: scope of the problem and nursing implications. Pediatr Nurs. (2008) 34(5):389–94. 19051842

[10] Johnson VR Acholonu NO Dolan AC Krishnan A Wang EHC Stanford FC. Racial disparities in obesity treatment among children and adolescents. Curr Obes Rep. (2021) 10(3):342–50. 10.1007/s13679-021-00442-0 33988825 PMC8120762

[11] Ogden CL Carroll MD Kit BK Flegal KM. Prevalence of obesity and trends in body mass index among US children and adolescents, 1999–2010. JAMA. (2012) 307(5):483–90. 10.1001/jama.2012.40 22253364 PMC6362452

[12] Stierman B, Afful J, Carroll MD, Chen TC, Davy O, Fink S https://stacks.cdc.gov/view/cdc/106273.

[13] Rimmer JH Yamaki K Lowry BMD Wang E Vogel LC. Obesity and obesity-related secondary conditions in adolescents with intellectual/developmental disabilities. J Intellect Disabil Res. (2010) 54(9):787–94. 10.1111/j.1365-2788.2010.01305.x 20630017

[14] Magaña S Lopez K Aguinaga A Morton H. Access to diagnosis and treatment services among Latino children with autism spectrum disorders. Intellect Dev Disabil. (2013) 51(3):141–53. 10.1352/1934-9556-51.3.141 23834211

[15] Zuckerman KE Lindly OJ Reyes NM Chavez AE Macias K Smith KN Disparities in diagnosis and treatment of autism in Latino and non-Latino white families. Pediatrics. (2017) 139(5):e20163010. 10.1542/peds.2016-3010 28557734 PMC5404727

[16] Magaña S Vanegas SB. Culture, race, and ethnicity and intellectual and developmental disabilities. In: Glidden LM Abbeduto L McIntyre LL Tassé MJ, editors. APA Handbook of intellectual and developmental disabilities: Foundations, Vol. 1. Washington, DC: American Psychological Association (2021). p. 355–82 (APA handbooks in psychology®)

[17] Bray GA Kim KK Wilding JPH, on behalf of the World Obesity Federation. Obesity: a chronic relapsing progressive disease process. A position statement of the world obesity federation: position paper. Obes Rev. (2017) 18(7):715–23. 10.1111/obr.12551 28489290

[18] Larsen JK Sleddens EFC Vink JM Fisher JO Kremers SPJ. General parenting styles and children’s obesity risk: changing focus. Front Psychol. (2018) 9:2119. 10.3389/fpsyg.2018.02119 30459686 PMC6232304

[19] Vaughn AE Ward DS Fisher JO Faith MS Hughes SO Kremers SPJ Fundamental constructs in food parenting practices: a content map to guide future research. Nutr Rev. (2016) 74(2):98–117. 10.1093/nutrit/nuv061 26724487 PMC4892304

[20] Lloyd AB Lubans DR Plotnikoff RC Collins CE Morgan PJ. Maternal and paternal parenting practices and their influence on children’s adiposity, screen-time, diet and physical activity. Appetite. (2014) 79:149–57. 10.1016/j.appet.2014.04.010 24751915

[21] Patrick H Nicklas TA. A review of family and social determinants of children’s eating patterns and diet quality. J Am Coll Nutr. (2005) 24(2):83–92. 10.1080/07315724.2005.10719448 15798074

[22] Pearson N Biddle SJH Gorely T. Family correlates of fruit and vegetable consumption in children and adolescents: a systematic review. Public Health Nutr. (2009) 12(2):267–83. 10.1017/S1368980008002589 18559129

[23] Yee AZH Lwin MO Ho SS. The influence of parental practices on child promotive and preventive food consumption behaviors: a systematic review and meta-analysis. Int J Behav Nutr Phys Act. (2017) 14(1):47. 10.1186/s12966-017-0501-3 28399881 PMC5387370

[24] Birch LL Fisher JO. Development of eating behaviors among children and adolescents. Pediatrics. (1998) 101(3 Pt 2):539–49. 10.1542/peds.101.S2.539 12224660

[25] Fleary SA Ettienne R. The relationship between food parenting practices, parental diet and their adolescents’ diet. Appetite. (2019) 135:79–85. 10.1016/j.appet.2019.01.008 30639293

[26] Puhl RM Schwartz MB. If you are good you can have a cookie: how memories of childhood food rules link to adult eating behaviors. Eat Behav. (2003) 4(3):283–93. 10.1016/S1471-0153(03)00024-2 15000971

[27] Mitchell GL Farrow C Haycraft E Meyer C. Parental influences on children’s eating behaviour and characteristics of successful parent-focussed interventions. Appetite. (2013) 60(1):85–94. 10.1016/j.appet.2012.09.014 23017468

[28] Power TG O'Connor TM Orlet Fisher J Hughes SO. Obesity risk in children: the role of acculturation in the feeding practices and styles of low-income Hispanic families. Child Obes. (2015) 11(6):715–21. 10.1089/chi.2015.0036 26584157 PMC4842941

[29] Wiley JF Cloutier MM Wakefield DB Hernandez DB Grant A Beaulieu A Acculturation determines BMI percentile and noncore food intake in Hispanic children. J Nutr. (2014) 144(3):305–10. 10.3945/jn.113.182592 24453127 PMC7720644

[30] Field D Garland M Williams K. Correlates of specific childhood feeding problems. J Paediatr Child Health. (2003) 39(4):299–304. 10.1046/j.1440-1754.2003.00151.x 12755939

[31] Sharp WG Berry RC McCracken C Nuhu NN Marvel E Saulnier CA Feeding problems and nutrient intake in children with autism spectrum disorders: a meta-analysis and comprehensive review of the literature. J Autism Dev Disord. (2013) 43(9):2159–73. 10.1007/s10803-013-1771-5 23371510

[32] Brzóska A Kazek B Kozioł K Kapinos-Gorczyca A Ferlewicz M Babraj A Eating behaviors of children with autism—pilot study. Nutrients. (2021) 13(8):2687. 10.3390/nu13082687 34444847 PMC8398283

[33] Schreck KA Williams K Smith AF. A comparison of eating behaviors between children with and without autism. J Autism Dev Disord. (2004) 34(4):433–8. 10.1023/B:JADD.0000037419.78531.86 15449518

[34] Leader G Tuohy E Chen JL Mannion A Gilroy SP. Feeding problems, gastrointestinal symptoms, challenging behavior and sensory issues in children and adolescents with autism spectrum disorder. J Autism Dev Disord. (2020) 50(4):1401–10. 10.1007/s10803-019-04357-7 31955310

[35] Mische Lawson L Bandy M Kadolph A Le A Pettersson S. The descriptive study of concerns of parents of children with ASD and factors related to obesity. Ther Recreation J. (2019) 53(2):117–31. 10.18666/TRJ-2019-V53-I2-9128

[36] Ausderau KK John BS Kwaterski KN Nieuwenhuis B Bradley E. Parents’ strategies to support mealtime participation of their children with autism spectrum disorder. Am J Occup Ther. (2019) 73(1):7301205070p1–7301205070p10. 10.5014/ajot.2019.024612 30839262 PMC6402415

[37] Odar Stough C Dreyer Gillette ML Roberts MC Jorgensen TD Patton SR. Mealtime behaviors associated with consumption of unfamiliar foods by young children with autism spectrum disorder. Appetite. (2015) 95:324–33. 10.1016/j.appet.2015.07.019 26206175 PMC4589473

[38] Roccatello G Cocchi G Dimastromatteo RT Cavallo A Biserni GB Selicati M Eating and lifestyle habits in youth with down syndrome attending a care program: an exploratory lesson for future improvements. Front Nutr. (2021) 8:641112. 10.3389/fnut.2021.641112 34568399 PMC8455913

[39] Ross CF Bernhard CB Surette V Hasted A Wakeling I Smith-Simpson S. Eating behaviors in children with down syndrome: results of a home-use test. J Texture Stud. (2022) 53(5):629–46. 10.1111/jtxs.12703 35696524

[40] Loth KA. Associations between food restriction and pressure-to-eat parenting practices and dietary intake in children: a selective review of the recent literature. Curr Nutr Rep. (2016) 5(1):61–7. 10.1007/s13668-016-0154-x

[41] Othman SI Fertig A Trofholz A Berge JM. How time in the US and race/ethnicity shape food parenting practices and child diet quality. Appetite. (2022) 171:105870. 10.1016/j.appet.2021.105870 34973995 PMC8996166

[42] Shloim N Edelson LR Martin N Hetherington MM. Parenting styles, feeding styles, feeding practices, and weight Status in 4–12 year-old children: a systematic review of the literature. Front Psychol. (2015) 6:1849. 10.3389/fpsyg.2015.01849 26696920 PMC4677105

[43] Tovar A Hennessy E Pirie A Must A Gute DM Hyatt RR Feeding styles and child weight status among recent immigrant mother-child dyads. Int J Behav Nutr Phys Act. (2012) 9:1–8. 10.1186/1479-5868-9-62 22642962 PMC3439673

[44] Hughes SO Power TG Orlet Fisher J Mueller S Nicklas TA. Revisiting a neglected construct: parenting styles in a child-feeding context. Appetite. (2005) 44(1):83–92. 10.1016/j.appet.2004.08.007 15604035

[45] LeCroy MN Siega-Riz AM Albrecht SS Ward DS Cai J Perreira KM Association of food parenting practice patterns with obesogenic dietary intake in Hispanic/Latino youth: results from the Hispanic community children’s health study/study of Latino youth (SOL youth). Appetite. (2019) 140:277–87. 10.1016/j.appet.2019.05.006 31063792 PMC6896789

[46] Jackson C Henriksen L Foshee VA. The authoritative parenting Index: predicting health risk behaviors among children and adolescents. Health Educ Behav. (1998) 25(3):319–37. 10.1177/109019819802500307 9615242

[47] Schmitz KH Lytle LA Phillips GA Murray DM Birnbaum AS Kubik MY. Psychosocial correlates of physical activity and sedentary leisure habits in young adolescents: the teens eating for energy and nutrition at school study. Prev Med. (2002) 34(2):266–78. 10.1006/pmed.2001.0982 11817924

[48] Lindsay AC Wallington SF Lees FD Greaney ML. Exploring how the home environment influences eating and physical activity habits of low-income, Latino children of predominantly immigrant families: a qualitative study. Int J Environ Res Public Health. (2018) 15(5):1–13. 10.3390/ijerph15050978 PMC5982017 29757941

[49] Suarez-Balcazar Y Agudelo Orozco A Mate M Garcia C. Unpacking barriers to healthy lifestyles from the perspective of youth with disabilities and their parents. J Prev Interv Community. (2018) 46(1):61–72. 10.1080/10852352.2018.1386270 29281599

[50] Taverno Ross SE Macia L Documét PI Escribano C Kazemi Naderi T Smith-Tapia I. Latino parents’ perceptions of physical activity and healthy eating: at the intersection of culture, family, and health. J Nutr Educ Behav. (2018) 50(10):968–76. 10.1016/j.jneb.2017.12.010 29954715 PMC6230483

[51] Lovasi GS Hutson MA Guerra M Neckerman KM. Built environments and obesity in disadvantaged populations. Epidemiol Rev. (2009) 31(1):7–20. 10.1093/epirev/mxp005 19589839

[52] Suarez-Balcazar Y Viquez F Miranda D Early A. Barriers to and facilitators of community participation among Latinx migrants with disabilities in the United States and Latinx migrant workers in Canada: an ecological analysis. J Community Psychol. (2020) 48(8):2773–88. 10.1002/jcop.22452 33016345

[53] Valdez Z Susana Ramírez A Estrada E Grassi K Nathan S. Community perspectives on access to and availability of healthy food in rural, low-resource, Latino communities. Prev Chronic Dis. (2016) 13(12). 10.5888/pcd13.160250 PMC5201142 27978407

[54] Hinckson EA Curtis A. Measuring physical activity in children and youth living with intellectual disabilities: a systematic review. Res Dev Disabil. (2013) 34(1):72–86. 10.1016/j.ridd.2012.07.022 22940161

[55] Ku B Rhodes RE. Physical activity behaviors in parents of children with disabilities: a systematic review. Res Dev Disabil. (2020) 107:103787. 10.1016/j.ridd.2020.103787 33017786

[56] Siebert EA Hamm J Yun J. Parental influence on physical activity of children with disabilities. Int J Disabil Dev Educ. (2017) 64(4):378–90. 10.1080/1034912X.2016.1245412

[57] Papadopoulos NV Whelan M Skouteris H Williams K McGinley J Shih STF An examination of parent-reported facilitators and barriers to organized physical activity engagement for youth with neurodevelopmental disorders, physical, and medical conditions. Front Psychol. (2020) 11:568723. 10.3389/fpsyg.2020.568723 33132976 PMC7550411

[58] Adeigbe RT Ramirez AG. Physical activity in Latino communities. NAM Perspect. (2015). National Academy of Medicine, Washington, DC. 10.31478/201504f Available from: https://nam.edu/perspectives-2015-physical-activity-in-latino-communities/ (Cited 2023 Mar 14).

[59] Nadon G Feldman DE Dunn W Gisel E. Mealtime problems in children with autism Spectrum disorder and their typically developing siblings: a comparison study. Autism. (2011) 15(1):98–113. 10.1177/1362361309348943 20484003

[60] Rhee KE Lumeng JC Appugliese DP Kaciroti N Bradley RH. Parenting styles and overweight status in first grade. Pediatrics. (2006) 117(6):2047–54. 10.1542/peds.2005-2259 16740847

[61] Quintero L. The needs and challenges experienced by Latino parents of children with developmental disabilities. Electron Theses Proj Diss. (2018) 734. Available from: https://scholarworks.lib.csusb.edu/etd/734

[62] Scherer N Verhey I Kuper H. Depression and anxiety in parents of children with intellectual and developmental disabilities: a systematic review and meta-analysis. PLoS ONE. (2019) 14(7):1–28. 10.1371/journal.pone.0219888 PMC6667144 31361768

[63] Pew Research Center. Hispanic Population and Origin in Select U.S. Metropolitan Areas, 2014. Pew Research Center’s Hispanic Trends Project.. Available from: https://www.pewresearch.org/hispanic/interactives/hispanic-population-in-select-u-s-metropolitan-areas/ (Cited 2023 Mar 14).

[64] Lukens CT Linscheid TR. Development and validation of an inventory to assess mealtime behavior problems in children with autism. J Autism Dev Disord. (2008) 38(2):342–52. 10.1007/s10803-007-0401-5 17578658

[65] Boles RE Scharf C Filigno SS Saelens BE Stark LJ. Differences in home food and activity environments between obese and healthy weight families of preschool children. J Nutr Educ Behav. (2013) 45(3):222–31. 10.1016/j.jneb.2012.09.012 23380192 PMC3640661

[66] Larios SE Ayala GX Arredondo EM Baquero B Elder JP. Development and validation of a scale to measure Latino parenting strategies related to children’s obesigenic behaviors. The parenting strategies for eating and activity scale (PEAS). Appetite. (2009) 52(1):166–72. 10.1016/j.appet.2008.09.011 18845197 PMC2659497

[67] CDC. All About Adult BMI. Centers for Disease Control and Prevention. (2022). Available from: https://www.cdc.gov/healthyweight/assessing/bmi/adult_bmi/index.html (Cited 2023 Mar 14).

[68] CDC. Growth Chart training resources.. Centers for Disease Control and Prevention. (2022). Available from: https://www.cdc.gov/nccdphp/dnpao/growthcharts/training/bmiage/page5_2.html (Cited 2023 Mar 14).

[69] IBM. Multiple Imputation. SPSS Statistics. (2021). Available from: https://www.ibm.com/docs/no/spss-statistics/26.0.0?topic=reference-multiple-imputation (Cited 2023 Mar 14).

[70] Tsoi MF Li HL Feng Q Cheung CL Cheung TT Cheung BMY. Prevalence of childhood obesity in the United States in 1999–2018: a 20-year analysis. Obes Facts. (2022) 15(4):560–9. 10.1159/000524261 35358970 PMC9421675

[71] Kahathuduwa CN West BD Blume J Dharavath N Moustaid-Moussa N Mastergeorge A. The risk of overweight and obesity in children with autism spectrum disorders: a systematic review and meta-analysis. Obes Rev. (2019) 20(12):1667–79. 10.1111/obr.12933 31595678

[72] Fryar CD Carroll MD Afful J. Prevalence of overweight, obesity, and severe obesity among adults aged 20 and over: United States, 1960–1962 through 2017–2018. National Center for Health Statistics (2021). Available from: https://www.cdc.gov/nchs/data/hestat/obesity-adult-17-18/obesity-adult.htm (Cited 2023 Mar 14). 41779887

[73] Campbell KJ Crawford DA Salmon J Carver A Garnett SP Baur LA. Associations between the home food environment and obesity-promoting eating behaviors in adolescence*. Obesity. (2007) 15(3):719–30. 10.1038/oby.2007.553 17372323

[74] Vereecken C Haerens L De Bourdeaudhuij I Maes L. The relationship between children’s home food environment and dietary patterns in childhood and adolescence. Public Health Nutr. (2010) 13(10A):1729–35. 10.1017/S1368980010002296 20883573

[75] Sirard JR Laska MN Patnode CD Farbakhsh K Lytle LA. Adolescent physical activity and screen time: associations with the physical home environment. Int J Behav Nutr Phys Act. (2010) 7(1):82. 10.1186/1479-5868-7-82 21078167 PMC2996341

[76] Van der Linde BW van Netten JJ Otten B Postema K Geuze RH Schoemaker MM. Activities of daily living in children with developmental coordination disorder: performance, learning, and participation. Phys Ther. (2015) 95(11):1496–506. 10.2522/ptj.20140211 26045605

[77] Blanco-Martínez N Delgado-Lobete L Montes-Montes R Ruiz-Pérez N Ruiz-Pérez M Santos-del-Riego S. Participation in everyday activities of children with and without neurodevelopmental disorders: a cross-sectional study in Spain. Children. (2020) 7(10):157. 10.3390/children7100157 33019630 PMC7600717

[78] Ogden CL Carroll MD Curtin LR McDowell MA Tabak CJ Flegal KM. Prevalence of overweight and obesity in the United States, 1999–2004. J Am Med Assoc. (2006) 295(13):1549–55. 10.1001/jama.295.13.1549 16595758

[79] Sun Y Supriya R Gao Y Yu S Wang A Ou X The relationships between parenting practices and child health-related behaviors in children with intellectual disability: the moderating role of child body weight Status. Nutrients. (2022) 14(24):5206. 10.3390/nu14245206 36558365 PMC9784023

[80] Prieto F Cabrera NJ Alonso A Ghosh R. Cultural and sociopolitical influences on African American and latinx fathers. In: Molloy S Azzam P Isacco A, editors. Handbook of the psychology of fatherhood. Cham: Springer International Publishing (2022). p. 239–60. 10.1007/978-3-031-14498-1_15

[81] Gorber SC Tremblay M Moher D Gorber B. A comparison of direct vs. self-report measures for assessing height, weight and body mass index: a systematic review. Obes Rev. (2007) 8(4):307–26. 10.1111/j.1467-789X.2007.00347.x 17578381

[82] Nordqvist. Why BMI is inaccurate and misleading. (2022). Available from: https://www.medicalnewstoday.com/articles/265215 (Cited 2023 Mar 11).

[83] Li W Liu Q Deng X Chen Y Liu S Story M. Association between obesity and puberty timing: a systematic review and meta-analysis. Int J Environ Res Public Health. (2017) 14(10):1266. 10.3390/ijerph14101266 29064384 PMC5664767

[84] Suarez-Balcazar Y Balcazar F Torres MG Garcia C Arias DL. Goal setting with Latinx families of children with intellectual and developmental disabilities: case studies. Behav and Soc Issues. (2022) 31(1):194–214. 10.1007/s42822-022-00094-2 PMC9018055 38625185

[85] Yin Z Liang Y Howard JT Errisuriz V Estrada VM Martinez C ¡ Míranos! a comprehensive preschool obesity prevention programme in low-income Latino children: 1-year results of a clustered randomised controlled trial. Public Health Nutr. (2023) 26(2):476–87. 10.1017/S1368980022002439 PMC10172390 36357340

